# Epigenetics in Critical Illness: A New Frontier

**DOI:** 10.1155/2013/503686

**Published:** 2013-07-09

**Authors:** Brian T. Graves, Cindy L. Munro

**Affiliations:** College of Nursing, University of South Florida, 12091 Bruce B. Downs Boulevard, MDC22, Tampa, FL 33612-4766, USA

## Abstract

Epigenetics is the study of alterations in the function of genes that do not involve changes in the DNA sequence. Within the critical care literature, it is a relatively new and exciting avenue of research in describing pathology, clinical course, and developing targeted therapies to improve outcomes. In this paper, we highlight current research relative to critical care that is focused within the major epigenetic mechanisms of DNA methylation, histone modification, microRNA regulation, and composite epigenetic scoring. Within this emerging body of research it is quite clear that the novel therapies of the future will require clinicians to understand and navigate an even more complex and multivariate relationship between genetic, epigenetic, and biochemical mechanisms in conjunction with clinical presentation and course in order to significantly improve outcomes within the acute and critically ill population.

## 1. Introduction

Critical care practice is beginning to look toward more specific cellular, biochemical, and genetic interventions in order to make a significant impact on patient outcomes. In addition to the extensive cellular, biochemical, and genetic body of research in process today, the science of epigenetics has become a more frequent focus within the critical care literature over the past 5+ years.

 Though epigenetics may appear to be relatively new to us in the critical care discipline, it has actually been studied for over 70 years and was first described by Conrad Waddington in 1942 as “the branch of biology which studies the casual interactions between genes and their products, which bring the phenotype into being” [1, 2]. In simpler terms, epigenetics is the study of changes in the function of genes that do not involve changes in the DNA sequence. It is the study of how the same sequence of DNA can produce significantly different phenotypes as a result of differing biochemical changes that alter gene availability for protein production [1, 3]. What makes this even more fascinating than a nature versus nurture discussion is that there are a small number of known genes in which specific biochemical modifications can impact the phenotype of offspring and are thus inheritable yet do not alter base pair sequencing of DNA. This is termed DNA imprinting [3]. A classic example of this is seen on chromosome 15 in Angelman and Prader-Willi syndromes where DNA methylation is involved in genomic imprinting of parental germ line cells, impacting the phenotype of the offspring depending upon whether the affected chromosome is paternal or maternal in origin [4–6]. Children with Prader-Willi syndrome inherit an affected paternal chromosome 15, resulting in short stature, poor muscle tone, and hypogonadism; many of these children also have learning disabilities. Children who inherit an affected maternal chromosome 15 may develop Angelman syndrome, which is associated with developmental delays, ataxia; they also may have epilepsy and microcephaly. 

Of even more importance to critical care, epigenetic changes of somatic cells can be propagated to progeny of those cells within an individual, impacting phenotypic expression during the course of critical illness. For example, epigenetic changes altering the effectiveness of immune cells to respond to pathogens could persist in new immune cells which inherited the epigenetic changes. These prior epigenetic changes could thus have a direct effect on an individual's ability to respond to sepsis in the future. 

 Interest in critical care has focused on DNA methylation, histone modification, and microRNA (miRNA). These epigenetic mechanisms can result in increased or decreased gene products. Decreased gene expression may result from downregulation of genes (the transcription of RNA from specific gene sequences is inhibited or arrested). Increased gene expression may result from upregulation of genes (increased transcription of RNA from targeted genes). However, for genes which provide a messenger RNA (mRNA) template for protein production, additional factors can influence the amount of protein produced from mRNA; for example, how often each mRNA is used for transcription and how quickly the mRNA is degraded can influence the amount of protein produced. More specifically, DNA methylation (as the result of enzymes known as DNA methylases) is the attachment of methyl groups (CH_3_) to cytosine bases within a DNA sequence; demethylation is removal of these methyl groups. As the quantity and pattern of DNA methylation increases, gene transcription into messenger RNA (mRNA) decreases; demethylation can increase gene transcription. Thus, DNA methylation represses expression of the affected genes. Methylation patterns in DNA can be transmitted to daughter cells during mitosis or transmitted to offspring as a result of meiosis [1, 7, 8]. 

**Figure 1:**
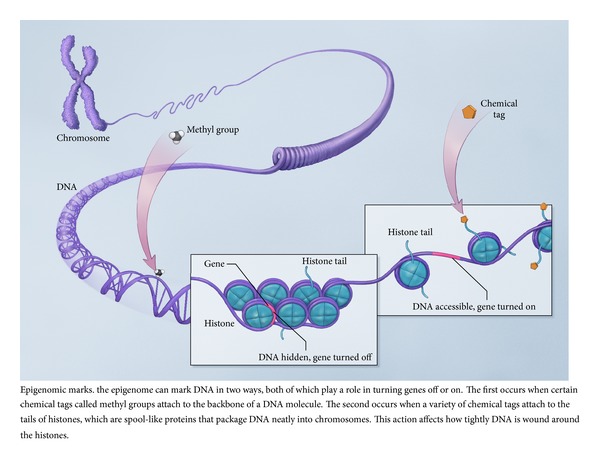
Epigenetics marks (courtesy: National Human Genome Research Institute) [10]. This figure is obtained from http://www.genome.gov/27532724.

Furthermore, since DNA is an extremely long molecule, it must be coiled and folded in order to fit into a nucleus (Figure 1). Histones are the nuclear proteins that direct the winding and coiling of DNA into nucleosomes and then chromatin. Histone proteins have extremely long tails which are susceptible to methylation, acetylation, ubiquitination, phosphorylation, and so forth at multiple locations [7]. When histone tails are modified (histone modification), they alter the way in which DNA will coil around a histone octamer (four histones with DNA coiled around them to form a nucleosome). The nucleosomes continue to fold and coil into chromatin, and multiple chromatin coils create a chromosome. In addition to gene sequence availability being limited by DNA methylation, how tightly chromatin is condensed will also impact the availability of gene sequences to interact with transcription proteins in order for mRNA to be transcribed and then translated into proteins [8, 9].

Epigenetic regulation can also occur through microRNAs (miRNAs). These small noncoding RNAs can act as regulatory elements in both transcription and translation. Noncoding RNAs are also involved in modifying phenotype through various mechanisms, such as posttranscriptional and transcriptional interference pathways, in which they may alter chromatin and/or DNA methylation processes to further stabilize gene silencing [1]. Roles for miRNAs in central nervous system injury and in acute lung injury have been postulated, although experimental evidence in critical care subjects is lacking. 

The extent of DNA methylation, histone modification, and microRNA activity may impact the function of genes without any alterations in the DNA sequence. These epigenetic mechanisms can have direct phenotypic implications. Several intriguing examples have recently been highlighted in critical care and are discussed below. 

## 2. Epigenetics in Critical Care

When gene expression is altered, the potential for significant phenotypical alterations to pathology, disease progression, and short- and long-term outcomes exists. Within critical care, research regarding the influence of genetics is in its early stages, and investigators are just beginning to look toward the science of epigenetics for explanations for patient and population differences in susceptibility to illness, clinical course, and outcomes. In the following sections, specific examples of epigenetic research focused on critical illness are provided.

### 2.1. Methylation of DNA

Epigenetic regulation, in which gene expression is altered and may significantly impact critical illness outcomes, can occur through direct methylation of DNA cytosine bases resulting in downregulation of genes. Alternatively, demethylation might upregulate expression of genes. An example of downregulation through methylation in acute illness has been associated with the pathological processes associated with acute kidney injury (AKI) [11]. 

AKI is a common complication in critical care patients, with an incidence greater than 5–10%, contributing to an increase in morbidity and mortality. Previous animal studies have suggested that altered expression of the KLK1 gene, which results in the transcription/translation of the serine protease kallikrein, may be related to AKI. Kallikrein is involved in the biochemical reaction in the kidney to produce kallidin, which pharmaceutically appears to have vasodilator and natriuretic properties in animals. Additionally, increased concentrations of kallikrein have been shown to be protective in animals, diminishing renal cell death by apoptosis and inflammation [11]. 

Kang and colleagues [11] prospectively compared hospitalized patients with established or incipient (early) AKI from ischemia, nephrotoxins, sepsis, and other causes to healthy nonhospitalized and ICU patient controls. In particular they were looking for the increased methylation of the promoter region of the KLK1 gene. The promoter region of a gene is a DNA sequence where the enzyme RNA polymerase binds to start mRNA transcription from the gene; the degree and pattern of methylation of promoters can regulate gene expression. Kang and colleagues hypothesized that gene silencing by methylation of the KLK1 gene promoter and thus the subsequent decrease of urine kallikrein may be associated with established AKI. Contrary to their expected findings, they found that established AKI patients, compared to controls, had significantly greater DNA methylation of KLK1 as expected but also had significantly higher levels of urine excreted kallikrein, which was not expected. Interestingly, the established AKI patients also had significantly lower average systolic blood pressure, increased heart rate, and increased epinephrine concentrations. Epinephrine is known to increase kallikrein concentrations in the urine, most likely as part of the systemic regulation of hemodynamic instability in acute illness. 

Two significant contributions to the state of epigenetic science in acute illness can be derived from the work of Kang et al. [11]. First, it further advances our understanding of how epigenetic modification may be trumped by other regulatory mechanisms. Second, this study provides an expanded perspective of the complexity of system regulation, highlighting the fact that even at the genetic level focusing on a single targeted therapy solution may need to give way to other more novel therapeutic approaches. 

### 2.2. Histone Modification

Another type of epigenetic mechanism, histone acetylation, is now a potential therapeutic target in critical illness. As previously discussed, DNA is more or less accessible for transcription depending upon how it is wound around histones in the nucleus. Acetylation of histones occurs when acetyl groups are added to specific amino acids (lysines) comprising the histones. Acetylation of histones changes the availability of the DNA in that area to transcription. Inhibitors of histone acetylation have been examined in animal models of hemorrhagic shock and LPS-induced sepsis; inhibiting histone acetylation reduces immune responsiveness during the acute episode, and has been associated with better outcomes [7]. Caution is warranted because not all cells respond similarly to pharmacologic agents targeted to histone modification, and acetylation inhibitors affect cellular proteins in addition to histones. Effects on nonhistone proteins may be positive, negative, or neutral.

Although histone modifiers are currently being explored as therapeutic agents, there is additional reason for caution because of emerging evidence about the sequella of histone modifications on immunity following sepsis. Patients who survive sepsis have profound and long lasting immunosuppression which can impede appropriate response to pathogens; 5- and 8-year survival is shorter compared to age-matched people who have not had severe sepsis. Evidence is accumulating that this consequence of critical illness is associated with epigenetic changes in immune cells. In a recent review of epigenetic mechanisms after sepsis, Carson IV and colleagues [7] provided several possible examples. Direct suppression of proinflammatory activity by epigenetic mechanisms has been hypothesized as cause for lipopolysaccharide (LPS, a major component of Gram-negative bacterial cell walls) tolerance. For example, in macrophages exposed to LPS (either in the laboratory or in a patient experiencing sepsis), an initial brisk proinflammatory response is followed by histone modifications to promoter regions of interleukin I beta and tumor necrosis factor alpha; these histone modifications reduce subsequent macrophage response to LPS resulting in immunosuppression. This has been demonstrated in animal models as well as in monocytes sampled from critically ill patients.

### 2.3. MicroRNA

MiRNA is highly expressed in central nervous system tissues, and research suggests that they play a role in neurodevelopment and neural plasticity [12]. Temporal alterations in expression of miRNAs localized at areas of central nervous system injury have been demonstrated in rodent models of spinal cord injury, traumatic brain injury, and brain ischemia [13, 14], although studies examining miRNA in human trauma victims have not yet been reported. Likely targets of these miRNAs have been identified by computational analysis and computer modeling based on sequence homology and include genes involved in inflammation and in neural signaling as well as other genes previously identified as important in response to specific central nervous system injuries. Madathil and colleagues [13] propose that in the response to acute CNS injury, some miRNAs might have a neuroprotective effect while others might have a neurotoxic effect. Thus, the epigenetic regulation mediated by miRNAs in the CNS is complex, and the effects in patients with CNS trauma or cerebrovascular accident will likely be difficult to be definitively determined.

MiRNAs contribute to differentiation and regulation of the immune system and have been implicated in chronic pulmonary diseases with an inflammatory component, including asthma, chronic obstructive pulmonary disease, and cystic fibrosis [15]. There has been recent speculation that miRNAs might also be involved in acute lung injury (ALI), including acute respiratory distress syndrome (ARDS, the most severe manifestation of ALI). Rodent models of ALI/ARDS, which initiate acute injury by administration of LPS, have provided some support for the involvement of miRNAs in the pathogenesis of ALI, but human data are not available. 

Better understanding of the positive and negative effects of miRNAs on the course of critical illness would be beneficial in at least two ways. First, it would help to elucidate mechanisms underlying pathogenesis and protective response; this could identify potential targets for pharmacotherapeutic or nonpharmacotherapeutic interventions. Second, in the future miRNAs could themselves be targets for intervention, with a goal of enhancing regulatory effects related to protective responses and suppressing regulatory effects associated with pathogenesis. 

### 2.4. Composite Scoring and Outcome Prediction

Warren and colleagues [16] investigated whether epigenetic data could improve on standard severity of illness scoring in trauma patients. Both Acute Physiology, Age, and Chronic Health Evaluation (APACHE) and Injury Severity Score (ISS) are well-validated tools which are widely used in clinical practice to score severity of illness [17, 18]. Building on the idea that gene expression changes early in trauma and critical illness might be prognostic of events occurring later in the hospital course, gene expression data could provide a snapshot predictive of likely outcome. In order to examine this concept, Warren and colleagues first developed a reference gene expression profile from the leukocytes of 10 healthy adults to calculate a reference score for each of 54,000 plus gene probe sets. Genomic response on the same gene probe sets of critically ill adult subjects within the first 12 hours of blunt trauma was compared to the healthy reference values by calculating difference from reference for each gene and then summing the differences, resulting in a difference from reference (DFR) score for each subject. They found that DFR scores, calculated early in the course of trauma, were positively associated with important clinical outcomes such as time on ventilator and length of stay. Since Warren and colleagues examined a single time point 12 hours after trauma, this study does not provide information about the magnitude of genetic expression changes over time nor when the optimal time to measure might be. 

A second group of investigators [19] refined this approach by retrospectively identifying 63 genes whose expression varied in trauma subjects over the course of 28 days of hospitalization and were significantly different between those with complicated and uncomplicated recovery. Of the 63 genes, two-thirds were related to protective immunity and a majority of those affected adaptive immunity. They found that a newly developed commercial multiplex system to rapidly quantify RNA (NanoString DFR, nanoString Technologies, Seattle, WA) was a better predictor of complicated outcome (versus intermediate or uncomplicated outcome) than standard microarrays, APACHE, or ISS score. Cuenca and colleagues [19] noted that current technologies are limited by the time required for processing samples; additional research into rapid technologies will be necessary. They further suggest that their data support a role for therapeutic agents that target adaptive immunity and gene expression data as indicators of likely response to biological response modifiers. 

Interestingly, temporal gene expression patterns in dendritic cells of 10 patients over the first four days following trauma identified upregulation of genes involved in antigen presentation [20]. However, the gene expression in dendritic cells was not associated with severity of illness scores. The differential expression of genes between subsets of cells (such as leukocytes and dendritic cells) and temporal changes in expression within a cell subset complicates the ability to develop predictive epigenetic measures of severity and outcomes. Analogous to the “left shift” in a white blood cell differential count, it may be essential to examine multiple genetics expression profiles from multiple cell types simultaneously to fully understand response. Important predictive patterns may emerge from such an approach, although technical considerations preclude this approach in the clinical setting at present. 

Stratification by genetic risk profile could inform when to initiate targeted therapy, who is most likely to benefit, and whether patients are responding to prescribed therapies. This information will be most useful if it can be assessed early in the course of critical illness. However, the specific epigenetic mechanisms underlying the changes in gene expression were not elucidated in the above studies [16, 19, 20]. 

## 3. Conclusion 

The state of the science in understanding the role of epigenetic regulation as it relates to the pathology of critical illness, clinical course, and outcomes is evolving rapidly yet still well within its infancy. Available research exists primarily within the lab, animal, and preclinical realm and thus has yet to be translated to the direct care of the critically ill person. Epigenetic tools and methodologies continue to evolve and improve, bringing the possibility of real time data to the point of care for therapeutic intervention closer to reality. Better identification and understanding of the role of epigenetic modifications that are associated with the complex regulatory and disregulatory processes of the disease state is essential and it is apparent that our approach to therapeutically target epigenetic modifications to improve outcomes may only be a single component of even more complex novel therapies to come.
